# A Comparison Between Two Different Approaches for a Collaborative Mixed-Virtual Environment in Industrial Maintenance

**DOI:** 10.3389/frobt.2019.00018

**Published:** 2019-03-27

**Authors:** Francesco De Pace, Federico Manuri, Andrea Sanna, Davide Zappia

**Affiliations:** Dipartimento di Automatica e Informatica, Politecnico di Torino, Turin, Italy

**Keywords:** augmented reality, virtual reality, mixed-reality, shared-reality, collaborative environment, interfaces, industry 4.0

## Abstract

Nowadays the market is becoming increasingly competitive, factories are required not only to enhance the product quality but also to reduce manufacturing and maintenance times. In an industrial context, modern factories are composed by many automated systems, such as industrial robots, which can perform different tasks. Although industrial robots are becoming more powerful and efficient, human workers are still required to accomplish different operations, such as training and maintenance procedures. The proposed research aims to assess a remote interaction system in an industrial training collaborative mixed-reality (CMR) environment. A remote expert user is capable of explaining a training procedure to an unskilled local user. Remote and local users interact using different interaction systems: the remote operator gives assistance using an immersive Virtual Reality (VR) device, whereas the local user interacts using a wearable Augmented Reality (AR) device. A comparison between an interaction based on the presence of a virtual human and one based on the use of abstract icons is proposed. In the first case, a virtual 3D representation of the remote technician is shown to the local user by using AR: the remote technician can pinpoint the components involved in the training procedure and the local user can visualize the instructions through some animations of the virtual avatar. In the second case, the local user cannot see a 3D representation of the remote technician; on the other hand, different 3D models, such as animated icons, are displayed to the local operator through AR depending on the component pinpointed by the remote technician in the virtual environment. Each 3D icon should suggest to the local user which component has to be manipulated at the current step of the procedure. Preliminary results suggest that the interface that requires less resources to be developed and managed should be preferred. Although in no audio condition the virtual avatar may improve the sense of presence of the remote technician, the use of abstract metaphors seems to be of primary importance to successfully complete an industrial task.

## 1. Introduction

Technology improvements are bringing new exciting opportunities to the industry domain. The fourth industrial revolution is changing how facilities work and how operators have to carry out their tasks. Since the market is becoming increasingly competitive, factories are required not only to enhance the product quality but also to reduce times and costs of training and maintenance procedures. Among the different available technologies and approaches, collaborative mixed-reality (CMR) systems can represent a reliable and innovative strategy to face the change of the fourth industrial revolution. Exploiting the characteristics and the capabilities of both VR and AR, industries can increase their quality and production improving the worker's performances. As the factories are becoming increasingly complex, operators are expected to be trained in shorter times without lowering the quality of the preparation. Using CMR tools, workers can benefit of the VR contents without losing the contact with the real world and without changing the way they work. The origin of these systems can be found in the first AR prototype proposed by Sutherland (1968). But it was not until the early years of the nineties that the underlying concepts of the Sutherland's innovation were formalized. Milgram and Kishino (1994) have introduced the concept of Mixed Reality as a continuum space going from full reality to full virtuality; AR display systems are part of this continuum and they give the possibility to augment the real world using computer generated features. Since AR and VR are parts of the same continuum, a connection between them can be indeed established and it can be employed for improving key operations performed in a factory, such as the training activities. Since operators should be trained using real objects in the real environment, the system proposed in this work will be evaluated from the AR point of view, comparing two different approaches, one based on the use of abstract virtual metaphors and one based on the presence of a virtual human avatar.

The paper is organized as follows: section 2 presents an overview of the use of the AR technologies in maintenance procedures. Section 3 introduces the proposed system along with the AR and VR interfaces. Section 4 shows the tests and the collected results. Section 5 illustrates the analysis and the evaluation of the results. Finally, conclusions and future works are presented in section 6.

## 2. State of Art

AR has been widely proved to be an effective tool in training operations that require manipulation of real objects, such as maintenance, repair and manual assembly. One of the first example of use of AR in an industrial application dates back to the nineties when Caudell and Mizell (1992) developed one of the first AR prototype to assist operators during assembly aircraft wire bundles procedures. Since then, several research groups and companies have been exploring the use of AR-based technologies in industrial applications. An example is the ARVIKA project (Friedrich et al., 2002), which mission was focused on the applicability of the AR tools in real scenarios. Researchers involved in ARVIKA found out that AR can be highly effective in the industry domain, reducing the development time and improving the overall production quality. Benefits of the use of the AR tools can also be discovered in the military domain: Henderson and Feiner (2009) developed an AR application that improves maintenance operations on an armored vehicle turret, proving that users were able to localize components 56% faster than when using traditional approaches. Small objects assembly operations are another field that benefits of the AR technology. In Baird and Barfield (1999), a comparison between an approach based on a small-scale assembly traditional procedure and an approach based on an AR tool is shown. Results demonstrate that participants completed the task faster and with fewer errors using the AR tool. Additionally, in Westerfield et al. (2015), an AR system was combined with an Intelligent Tutoring System (ITS) to assist operators during a motherboard assembly procedure. Authors proved that the task performance was 30% faster compared to the same AR training system without intelligent support. AR can also be applied in maintenance procedures, that is one of the core activity of the industrial production life-cycle since it accounts for as much as 60–70% of its total costs (Mourtzis et al., 2016). In Manuri et al. (2014), authors illustrated how to design and develop AR applications to support industrial maintenance with particular interest for the markerless tracking technology. Moreover, in Sanna et al. (2015a), a comparison between an interaction based on AR technology and one based on canonical paper instructions has been evaluated. Results suggest that the lower is the skill of the users, the greater is the effectiveness of the AR technology.

Thanks to the technological improvements, nowadays it is possible to exchange large amount of data on long distances with low latencies. Companies are increasingly interested in the development of technologies that allow collaborative maintenance and training procedures and several works have investigated the use of AR in remote assistance systems (Zhong et al., 2002; Ou et al., 2003; Fussell et al., 2004; Sakata et al., 2006; Alem et al., 2011; Benbelkacem et al., 2011; Chen et al., 2013; Kim et al., 2013; Wang J. et al., 2014). In Bottecchia et al. (2010), a remote tele-assistance AR system has been developed: a remote skilled operator can help a local user, indicating the objects to be used during the maintenance procedure. The local user wears an AR glasses that records the real environment and the corresponding streaming is sent to the remote user. Then, the remote user can add annotations and abstract symbols on the frames by clicking on the surface of the visualization device. Moreover, an audio channel is provided allowing users to exchange information in real-time. In Mourtzis et al. (2017), a smart assembly/disassembly algorithm for automated generation of assembly sequences is combined with the possibility for a local user to visualize AR maintenance instructions provided by a remote expert technician. The application has been tested on a real maintenance case, which consists in a battery pack replacement of an industrial robot. Starting from an initial procedure cost of € 1,370 and a completion time of 9 h, the AR tool has reduced the overall cost to € 150 and the completion time of 2 h. Sanna et al. (2015b) proposed an AR maintenance collaborative system that allows a skilled remote operator to modify the virtual assets and instructions in real-time to give assistance to a local operator. The maintenance procedure is represented by a finite state machine composed by nodes and arcs. Each node represents a particular step of the procedure and it contains all the virtual aids used by the remote operator. Arcs contain the tracking information and they consist of a CAD model representing the real object manipulated by the local operator. Results indicate that the number of errors made during the procedure was reduced with the support of the remote operator, lowering also the differences in time spent by the local users to accomplish the task.

Besides using abstract metaphors to support an unskilled operator, several additional works have tried to improve the perception and the efficiency of the collaboration among users adding human gestures to the augmented scene (Goto et al., 2010; Tecchia et al., 2012; Sodhi et al., 2013; Wang X. et al., 2014; Yin et al., 2014; Huang et al., 2018). In Yin et al. (2014), pre-animated virtual hands show the user how to perform a manual operation on an industry product in an AR scenario. Results demonstrate that displaying a human body part that performs the maintenance task improves the user learning of the procedure and it allows the users to comprehend the sequence of operations in an intrinsic and natural way. The recent technological improvements in reconstruction and human motion tracking have allowed to create animated and realistic virtual avatars and considerable efforts are devoted to understand how human beings react in presence of virtual agents. As confirmed by several recent works, such as Jo et al. (2015, 2017) and Koskela et al. (2018) or commercial applications^1^, the different uses of a virtual avatar are increasingly becoming object of interest and analysis. To make sure that the avatar controlled by the remote expert operator will be positively accepted by the local unskilled user, the behavior of the virtual agent and its position in the real world should be as realistic and convincing as possible. Thus, it becomes of primary importance, for the remote operator, to analyze the local operator environment from an independent point of view, as explored in Tait and Billinghurst (2014, 2015). Their results suggest that when the remote operator is capable of analyzing and interacting in the scene independently from the local operator's point of view, tasks are completed faster and with more confidence from the users. More recently, Wang et al. (2019) have presented an AR system in which the remote operator is able to move independently from the local operator's position, allowing the remote operator to visualize the working space and to offer assistance from different points of view. Overall, the state of the art highlights the relevance of two aspects, which can improve the assistance to the local user and that are strictly related: firstly, the effectiveness of the AR animations, which could be enhanced by adding to the scene an avatar representation of the remote technician; secondly, the importance of the view independence for the remote technician.

The proposed research aims to investigate how these aspects could enhance the collaboration in a CMR environment: this is made by deploying a prototypal, mixed-reality collaboration system based on a shared environment, where the remote technician is represented by an avatar and his/her movements and interactions are provided in real-time to the local user by AR. The proposed system allows the remote operator to provide assembly instructions acting independently in an Immersive Virtual Environment (IVE), while the local operator can benefit of the virtual contents as augmented instructions, related to object in the real environment, using an AR device. A comparison between an interaction based on a virtual human presence and one based on the use of abstract icons is proposed to understand whether the presence of a human avatar can effectively improve the learning ability of the local user in an industrial training procedure.

## 3. The Proposed System

The first step to develop the proposed system consists in defining the design requirements. Starting from the analysis of the state of the art, a typical scenario of an assisted maintenance procedure consists of:

- A user performing a task in a dedicated physical environment, comprehensive of tools, components to perform the task and the object to be maintained;
- A set of instructions to help the user in completing the task; the most traditional case consists of paper manuals, whereas recent solutions consist of AR applications, which provide instructions as augmented reality contents;
- A way to communicate to an expert in order to ask for help if the user cannot understand how to perform the task solely from the instructions; this can consist of a simple phone/video call or a sophisticated shared environment;

In order to compare the two distinct interfaces (abstract metaphors and avatar) in a mixed reality environment, a shared environment has been designed. A local operator (trainee) has to be trained to perform a task by a remote operator (trainer). Physically, the trainee and the trainer are not located in the same environment. The trainee's real environment comprehends a set of tools and objects that are needed to perform the task and the trainee can access the shared environment through AR to receive instructions from the trainer. The trainer accesses the shared environment through an Immersive VR interface. In this way, the trainer does not need a local replica of the real environment but he/she can interact with a virtual representation of the object of interest in the real environment. Finally, the system allows the two users to communicate through a bidirectional audio channel. Based on the proposed goal, there are two important aspects to be considered above all: firstly, the coordinate systems, which means how the coordinates of the objects in the shared environment are exchanged between the virtual and augmented environments; secondly, the virtual elements used to assist the trainee, which comprehend all the virtual objects, animations and the avatar representation. Since in the AR environment all the virtual elements are aligned with respect to a known target, it is indeed reasonable to use the known target frame as a shared reference system in both environments. Thus, all the virtual elements can be correctly aligned in both worlds. The virtual elements used in maintenance procedures can be represented by abstract metaphors (such as lines, shapes, arrows, etc.) or by using a virtual avatar. These virtual assets can be used to accomplish at least two different actions: pinpointing to a specific object and/or showing how the object should be manipulated. The pinpoint action can be either expressed using 3D arrows or shapes placed at the object's location or it can be executed by the avatar itself. The objects manipulation can be shown by an animated version of the 3D objects themselves or by an animated avatar that shows how the objects should be manipulated by the trainee. In this work a set of pre-computed animations has been used to present the virtual avatar movements; this choice is due both to the requirement to guarantee the same visualization to all trainees and to the lack of a real-time tracking system to measure the trainer's movements. The system should allow the trainer to see, in the virtual world, a room that contains the position of both the trainee and the objects involved in the maintenance procedure. Whereas the objects' position is considered previously known, the trainee's position should be updated in real-time. Finally, the trainer should be able to interact with the objects in the scene and to highlight points of interest to the trainee. The trainee instead should be able to see the AR animations correctly aligned in the real environment.

**Figure 1:**
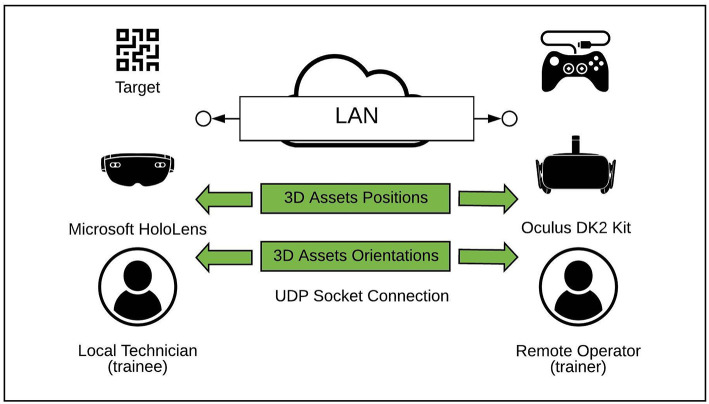
The system architecture.

The System Architecture of the proposed environment is illustrated in Figure 1. The Hardware architecture is composed by two different devices connected on the same Local Area Network (LAN). The trainer interacts in the VR environment through an Oculus Rift DK2 Kit and a Microsoft XBOX 360 gamepad: this configuration allows the trainer to support the trainee from a desktop station. More specifically, the Oculus Rift provides the trainer with an immersive view of the shared environment, whereas the gamepad allows the trainer to move in the virtual environment and interact with it. Concerning the trainee, a wearable device, the Microsoft HoloLens^2^ glasses, has been preferred to a solution based on handheld or projected devices. Hence, the trainee is capable of visualizing 3D virtual contents keeping hands free to perform any possible task. The shared environment has been realized as a Unity3D application. The Oculus Rift DK2 acts as a server, whereas the HoloLens glasses acts as a client. In addition to the Unity3D Integrated Development Environment (IDE), some libraries and APIs have been used to manage several aspects of the application. The most relevant are:

- The SteamVR Plugin^3^ to access the Oculus Rift DK2 hardware;
- The Unet Unity API^4^, to manage the multi-users architecture (specifically the High Level API);
- The Vuforia^5^ library to track a physical target, to correctly align the different environments.

Since the same application is used both for the VR and the AR environments, the same project has been built for two distinct target platforms, the *Universal Windows Platform* for the AR device and the *PC, Mac, and Linux Standardize* platform for the VR hardware. No open-source, mixed-reality project or framework have been found compliant with both the selected hardware and the design choices, thus, the prototypal, mixed-reality system has been developed from scratch.

### 3.1. Use Case

To compare the two interfaces, a training task has been chosen. The task consists of assembling the T42 3D printed hand (Odhner et al., 2013), developed by the Yale School of Engineering and Science (the .step and .stl files are freely available to download^6^). The complete real pieces list and the procedure for assembling the T42 hand can be found online^7^. Although the 3D printed hand can be seen as a simplified version of a real industrial robot hand, it is certainly related to the industrial robotic area and thus it can be reasonably used to train a robot technician. Its relative simple design and the use of non-hazardous materials assure to be used and tested by unskilled users, not trained for industrial procedures. Furthermore, the availability of the files ensures the repeatability of the proposed experiment.

**Figure 2:**
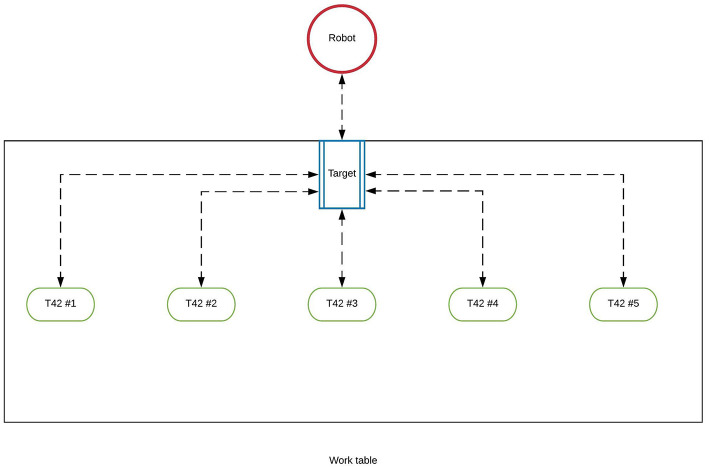
The T42 hand pieces and the industrial manipulator have been positioned in some predefined locations respect to the target.

Since the entire assembling procedure requires a huge amount of time to be completed, only a subset of the hand pieces has been used (Table 1). The models have been 3D printed using two different 3D printers to speed up the printing process: the Snapmaker 3D printer^8^ and the Anycubic i3 Mega. By following the procedure given by the trainer, the trainee's goal is to assemble the hand and to place it on custom 3D printed flange (also available at the Yale repository) attached to an industrial robot. In order to correctly synchronize the positions and orientations of the real hand pieces and of the robotic arm within the virtual environment, they have been placed at some predefined positions respect to the target (Figure 2).

**Table 1:** The list of the components used to assemble the T42 robotic hand.

**Name**	**Quantity**
a1_p_t42.STL	1
a4_coupling_t42.STL	1
c1_t42.STL	2
finger_pp_B_t42.STL	2
finger_pp_ext_A_t42.STL	2

### 3.2. Interfaces

Both the trainee and trainer can visualize a virtual representation of the other operator in real-time. When the trainer is moving in the virtual environment, the same motion is applied to a 3D avatar of the trainer in the AR scenario. The same concept is suitable for the motion of the trainee: when the trainee detects the image target, the position and orientation of the trainee are calculated to place the corresponding virtual avatar in the trainer'scenario. Thus, when the real trainee is moving around the environment, his/her 3D representation is correctly moving in the trainer scenario. The difference between the application with the virtual avatar and the one with the abstract metaphors resides in the way graphical instructions are conveyed to the trainee.

This work focuses on the evaluation of two AR interfaces for supporting operators in training procedures. Although the VR interface is briefly presented, its evaluation is out of the scope of this paper as it just allows the trainer to play pre-computed animations related to objects pointed by the gaze (this version of the system does not provide a real-time tracking system to track the trainer movements). Moreover, several works have already investigated Immersive VR interfaces for maintenance operations and interested readers can find more details in McNamara et al. (2016), Linn et al. (2017), Louison et al. (2017), Eschen et al. (2018), and Guo et al. (2018).

#### 3.2.1. The AR Interfaces

Two original distinct AR interfaces have been developed for this work. They differ only for some specific 3D contents, the abstract metaphors and the 3D avatar. Table 2 summarizes both interfaces.

**Table 2:** The 3D models of both AR interfaces.

**Abstract metaphors**	**Virtual avatar**
3D arrow	Avatar VR
3D cursors	3D cursors
3D hand pieces	3D hand pieces

**Figure 3:**
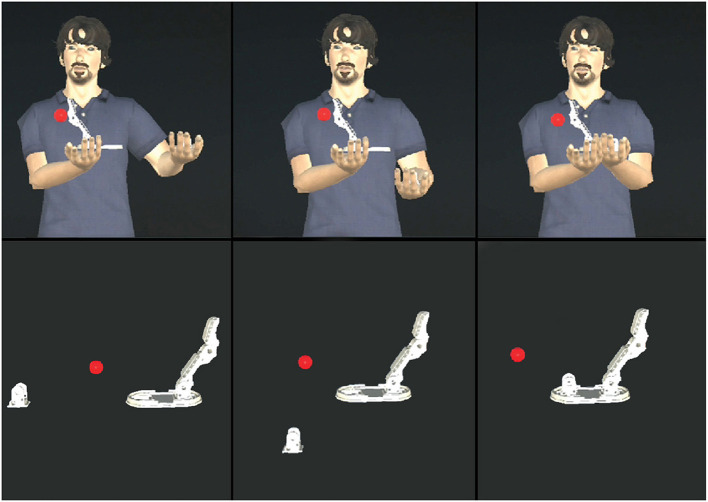
First row: from left to right, one of the assembly animations played by the virtual avatar. Second row: the same animation played without the virtual avatar in the abstract AR interface.

The 3D cursors consist of small 3D red spheres that are rendered at the coordinates of the user's sight. To achieve this behavior, a ray-cast is performed starting from the center of the virtual camera. Then, when the ray-cast hits a 3D model, the cursor is rendered on the collision coordinates. The virtual abstract metaphors are represented by 3D arrows. When the trainer points to a specific 3D model in the VR environment, a virtual arrow is placed at the pointed position. The 3D avatar consists in a virtual representation of a worker. To supply an effective assistance, some animations have been added both to the virtual T42 hand pieces and to the virtual avatar. Moreover, the addition of the animations to both interfaces assures to fairly compare the two interfaces, avoiding giving more capabilities to one interface respect to the other. In the abstract metaphor scenario, the animations of the pieces show how to correctly combine them, whereas, the animations of the 3D avatar himself show the trainee how to correctly combine the pieces (Figure 3). Moreover, to improve the realism of the virtual avatar, three other types of animations have been added to the 3D avatar: an idle animation, a walking animation and a hand pointing animation. The rigging and animation procedures have been done using the Mixamo^9^ tool that allows to add pre-defined animations to humanoid characters. The pre-defined animations have been employed for two main reasons: firstly, the assembly procedure is composed by pre-determined steps and secondly the use of pre-calculated animations ensures to visualize the animations of the virtual avatar always in the same manner, allowing to fairly analyze the effectiveness of the AR interface. All the animations are played when the trainer presses the corresponding controller buttons, except for the pinpointing animation that is applied in two different steps. Firstly, when the trainer presses the pinpointing animation button, a check on the ray-cast is performed. If the ray-cast actuated in the virtual environment returns a coordinate in the 3D space, the coordinate is used to apply an inverse kinematic algorithm on the right arm of the avatar to represent the pinpointing movement. Finally, the hand pointing animation is played.

#### 3.2.2. The VR Interface

**Figure 4:**
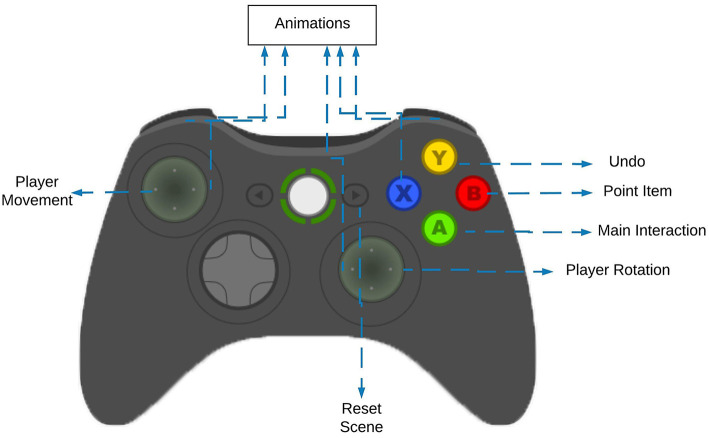
The description of the controller.

A new VR interface has been developed to grant the interaction of the trainer. It is essentially composed by two different layers: the tangible and the gaze layers. The first one regards the physical input given by the user. Since the Oculus DK2 does not provide any form of interaction, a XBOX 360 gamepad has been added to the system to provide a proper interaction interface. As it is possible to notice from Figure 4, the left and right analog sticks are used to translate and rotate the trainer respectively, whereas the B button is used to pinpoint the 3D models. In order to ensure an effective selection system, a gaze interaction mechanism has been added. Since the Oculus DK2 consists of a 6 degrees of freedom device, the user is able to look around the environment in all the possible directions, hence adopting the integrated gaze system provided by the Oculus it is possible to determine which object the user wants to operate with. Although this layer implementation is similar to its AR counterpart, the interaction paradigm results to be quite different: if the trainer presses the B button while looking at a specific 3D model, the 3D model becomes selectable and manipulable. The combination of the tangible interface with the gaze one should ensure a reliable and natural interaction mode.

**Figure 5:**
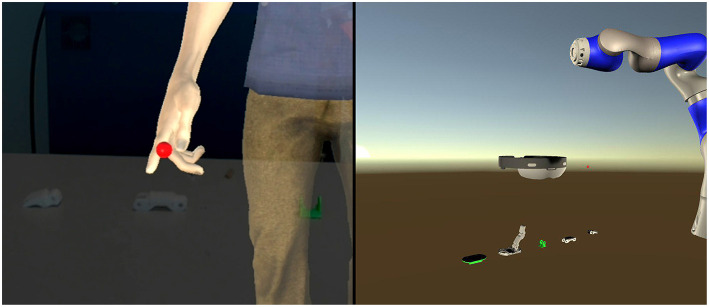
**Left**: the AR avatar interface. The virtual avatar is pointing to a real hand's piece. **Right**: the VR interface. The AR avatar is represented by a virtual representation of the Microsoft HoloLens glasses.

The trainer can visualize and interact with several typologies of 3D models (Table 3). A subset of them has been already discussed in the previous chapter, thus only the latter are introduced in this section. The virtual robot is represented by a collaborative manipulator. Since only the data relative to the position and orientation of the Microsoft HoloLens glasses are shared in the shared environment, it has been possible to represent only the head of the trainee. Nevertheless, the combination of the gaze layer with the data relative to the position and orientation of the HoloLens results to be suitable to understand the position of the trainee and where he/she is gazing. Figure 5 shows the AR avatar interface and the VR one.

**Table 3:** The 3D models of both VR interfaces.

**VR interface**
3D cursors
3D hand pieces
Virtual manipulator
Avatar AR

In the following section, the interaction system will be discussed.

### 3.3. The Interaction System

**Figure 6:**
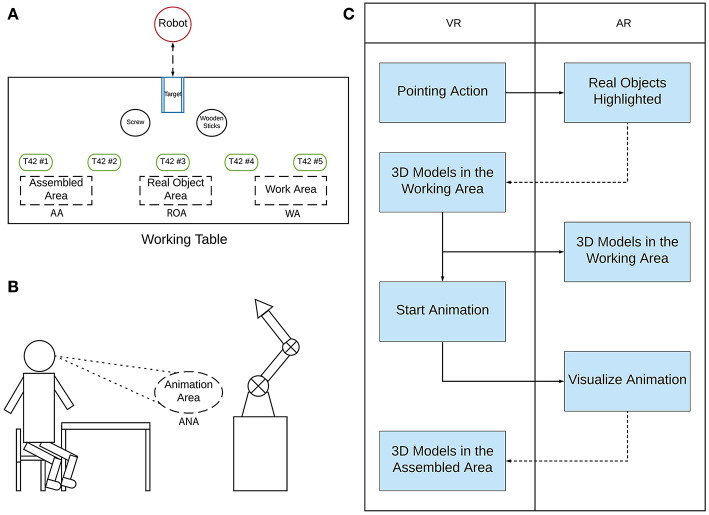
Interaction work-flow. Top-left image **(A)** represents the ROA, WA, and AA areas. The bottom-left image **(B)**: the animation area. The right image **(C)**: the interaction workflow.

The operative area of the trainee has been divided into three different zones: the real objects' area (ROA) the working area (WA) and the assembled area (AA) (Figure 6A). In the abstract metaphor interface, all the animations of the 3D models appear in front of the user (Figure 6B), within the animation area denoted by ANA. Only the animation representing the final step of the procedure behaves differently because it is played at the end-effector position of the manipulator. On the other hand, by using the interface with the virtual avatar, the animations are played by the character itself at its position.

In Figure 6C, it is possible to visualize the workflow interaction. At the beginning, when the trainer selects one of the 3D models, the corresponding real hand piece is highlighted in the ROA of the trainee. Once the trainee gives a positive feedback to the trainer by the audio channel, the trainer moves the selected 3D model in the WA allowing the trainee to clearly understand if the picked is the right one. Then, the trainer plays the corresponding animation to assemble the hand's pieces and the trainee visualizes it in the ANA. Finally, once the trainee confirms to have completed the procedure step, the assembled piece is rendered in the AA.

## 4. Tests and Results

In the following sections, the tests and results are presented.

### 4.1. Tests

In order to compare the two different modalities of training, some tests have been carried out at Politecnico di Torino. Twenty students have been identified, with ages that ranged between 20 and 28 years. Participants were all volunteers and they gave written informed consent in accordance with the Declaration of Helsinki. All tests have been conducted in compliance with the ethical code defined by the article 2, paragraph 4 of the Italian law 240, issued on the 30 December 2010. Ethical approval was not required in line with the aforementioned legislation. Users had to try to build the T42 hand following the instructions of the remote trainer. Since the comparison is evaluated only from the AR point of view, the figure of the trainer has been interpreted by one of the paper's author. Testers have been divided into two different groups (called A and B): tests of A group are focused on analyzing the abstract metaphors interface whereas tests of group B are focused on analyzing the virtual avatar-based interface. Tests have been accomplished following the subsequent procedure:

1. Users have been introduced to the test. Specifically, they have been informed of the fact that a remote operator would have explained to them how to build the real T42 hand;
2. Users of both groups have tested the corresponding interface;
3. After the test, a questionnaire has been proposed to the users.

Two questionnaires have been prepared, called Questionnaire A (QA) and Questionnaire B (QB). Both QA and QB are divided in three different sections: the first one regarded the user's information and his/her knowledge of AR whereas the second section is composed by nine different statements (Polvi et al., 2013). The third section of both QA and QB consists of eleven statements. The 11 statements in QA regard the abstract metaphors, whereas the statements of QB concern the virtual avatar. The statements relative to the second and third sections were ranked in 5-point Likert scale (from 1 = strongly disagree to 5 = strongly agree). The questionnaire also included an open text question for free comments. Finally, time completion and number of errors have been recorded: an error took place if the user positioned a piece in the wrong position or with the wrong orientation at a given step of the procedure.

**Figure 7:**
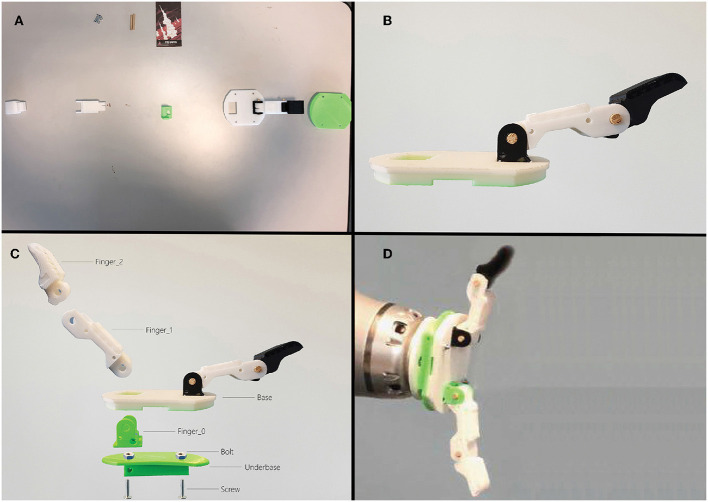
Top-left image **(A)**: the initial configuration. Top-right image **(B)**: the finger already assembled. Bottom-left image **(C)**: the renamed hand pieces. Bottom-right image **(D)**: the complete hand placed at the end-effector position.

The initial configuration of the real hand pieces is illustrated in Figure 7A. One of the two fingers was already assembled and inserted into the base (Figure 7B). Hence, users had to complete the other half of the 3D printed hand, assembling the latter finger and plugging it into the base. Further, users were suggested to use the half assembled hand as reference for better understanding the orientation of the pieces. In order to complete some specific steps of the assembling procedure, additional material has been provided to the testers: two tiny wooden sticks, two screws and two bolts. The real pieces and the robot were positioned at some predefined distances from the target. At the beginning of the procedure, users had to sit down on a chair positioned in front of a table. Then, after having assembled the robotic hand, testers had to plug it on a 3D printed support placed on the end-effector of the industrial manipulator (Figure 7D).

The complete procedure consists of the following steps (see Figure 7C to understand the renamed hand pieces):

1. Take the Finger_0 and the Base;
2. Plug the Finger_0 into the Base (new piece called F_Base);
3. Take the Finger_1 and the Finger_2;
4. Combine the Finger_1 and the Finger_2, using the wooden sticks (new piece called Finger);
5. Take the F_Base and combine it with the Finger, using the wooden sticks (new piece called Hand_1);
6. Take the Underbase and attach it to the Hand_1, using the two screws and the two bolts (new piece called Hand_2);
7. Plug the Hand_2 on the manipulator's end-effector.

**Figure 8:**
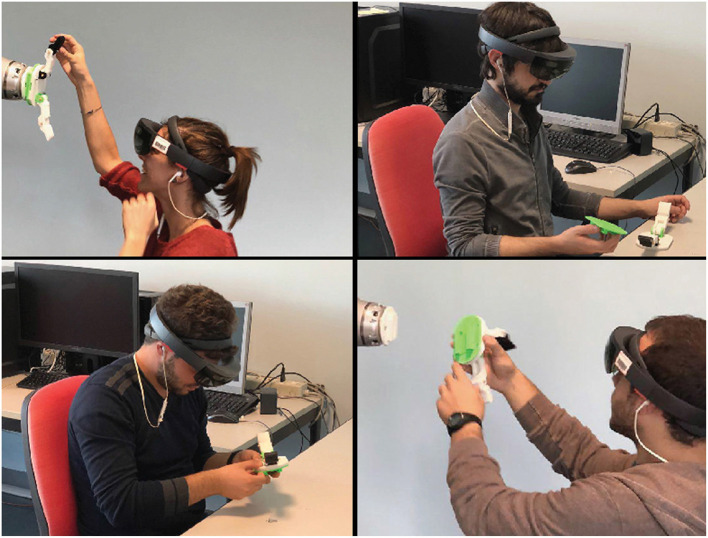
Users evaluating the multi-users mixed reality environment.

In order to supply a feedback mechanism, an audio channel has been established between the two users using two smartphones and two Bluetooth earphones. To ensure that all the users were able to receive the same instructions, a text file has been prepared with the instructions that the trainer had to provide to the AR users for each step of the procedure. Figure 8 shows some users following the training procedure.

### 4.2. Results

The results aggregation of the first section of the questionnaires is shown in the first part of Table 4. Despite almost all the testers declared to know AR, just over half the participants had tried an AR application before. Moreover, all the users declared that they did not know the T42 robotic hand before tests. The second section of the questionnaire has been evaluated considering the mean (M) and the standard-deviation (SD). As can be noticed from the second part of Table 4, both systems have obtained positive responses, except for the statement A2, that was negative worded. In all the statements, the abstract metaphor-based interface has obtained higher values than the virtual avatar system but the last two assertions. For what concern SD, results of the abstract metaphors are less distributed, showing more uniform answers. The third section was evaluated using two different methodologies. Table 5 shows the collected results: the first half of the table refers to the abstract metaphors whereas the second concerns the virtual avatar. Firstly, the M and SD values have been computed for each declaration. Statements Q4/Q15, Q6/Q17 and Q9/20 were negative worded. Overall, the M values of the abstract interfaces are marginally superior to the ones of the avatar interface. Moreover, even if the abstract interface's SD is lower than the avatar interface's one, its SD results to be considerably high for almost all the assertions of both interfaces. Secondly, an unpaired *t*-test (*p* = 0.05) has been computed on the third section of the questionnaire. The null hypothesis proposed in this work is the same for all the statements and it can be expressed as follows: “*The virtual avatar-based interface outperforms the abstract metaphor-based one.”* The same questions expressed in the two different questionnaires have been paired to compute the *p* value. As the *p-value* is clearly above the threshold value of 0.05 for all the statements, it is not possible to declare the results statistically significant and the null hypothesis has to be rejected. Finally, for what concerns the time completion, no significative differences have been found between the two interfaces.

**Table 4:** Answers of the first and second sections of the questionnaire.

**#**	**Questions**				
1	Age	Average = 24.5			
2	Gender	70% Male		30% Female	
		**Yes**		**No**	
3	Do you know what Augmented Reality is?	90%		10%	
4	Have you ever used an Augmented Reality application?	65%		35%	
5	Do you know the Yale Model T42 Robotic hand?	0%		100%	
6	Have you ever assemble the Yale Model T42 Robotic hand?	0%		100%	
		**Metaphors**		**Avatar**	
		**M**	**SD**	**M**	**SD**
A1	I think this system was easy to use. (Efficiency)	4.4	0.51	4.2	0.63
A2	I would need the support of a technical person to be able to use this system. (Learnability)	1.7	1.05	1.7	0.94
A3	The user interface of this system is pleasant. (User satisfaction)	3.6	0.51	3.7	0.67
A4	I can effectively complete my tasks using this system. (Efficiency)	5	0	4.8	0.42
A5	This system gives me clear instructions. (Learnability / Efficiency)	4.9	0.31	4.5	0.52
A6	It was easy to learn how to use this system (Learnability)	4.9	0.31	4.7	0.67
A7	I would recommend this system to my friends or colleagues. (User satisfaction)	4.7	0.48	4.4	0.69
A8	The feedback given by this system is easy to understand. (Learnability / Efficiency)	4.3	0.67	4.5	0.71
A9	Overall, I am satisfied with this system. (User satisfaction)	4.3	0.48	4.5	0.52

*M, mean value; SD, standard deviation*.

**Table 5:** Answers of the third section of the questionnaire.

**#**	**Questions**	**Metaph (M)**	**Metaph (SD)**
Q1	The 3D arrows have clearly indicated the required real pieces to use during the procedure.	4.4	0.69
Q2	The animations of the 3D models have clearly shown how to combine the real objects.	4.6	0.69
Q3	It seemed to me to collaborate with the remote person.	4.6	0.69
Q4	It seemed to me to work alone.	1.4	0.69
Q5	It seemed to me to be in the same room with the remote person.	3.9	1.19
Q6	It seemed to me to be alone in the room.	1.9	1.28
Q7	The animations and the 3D arrows have clearly shown how to plug the hand on the end-effector of the robot.	4.6	0.69
Q8	I was able to complete the procedures without watching several times the animations.	4.8	0.41
Q9	I needed to repeate the procedures several times.	1.1	0.31
Q10	The audio instructions have been fundamental to complete the procedure.	3.7	1.15
Q11	I think I could complete the procedure without the audio instructions.	2.9	1.10
**#**	**Questions**	**Avatar (M)**	**Avatar (SD)**
Q12	The virtual avatar has clearly indicated the required real pieces to use during the procedure.	4	0.66
Q13	The virtual avatar has clearly shown how to combine the real objects.	4.6	0.15
Q14	It seemed to me to collaborate with the remote person.	4	1.15
Q15	It seemed to me to work alone.	2	1.33
Q16	It seemed to me to be in the same room with the remote person.	3.5	1.51
Q17	It seemed to me to be alone in the room.	2.1	1.45
Q18	The animations of the virtual avatar have clearly shown how to plug the hand on the end-effector of the robot.	4.4	1.26
Q19	I was able to complete the procedures without watching several times the animations.	4.5	0.71
Q20	I needed to repeate the procedures several times.	1.5	0.08
Q21	The audio instructions have been fundamental to complete the procedure.	4.1	1.10
Q22	I think I could complete the procedure without the audio instructions.	2.8	1.13

*M, mean value; SD, standard deviation*.

In the following section, the analysis of the results is presented along with their comments.

## 5. Results Analysis

Considering the M and SD values, the two interfaces can be deemed only as comparable. Despite these outcomes, some observations and evaluations are possible. Although it was not possible to prove that one interface outperforms the other one, it is reasonable to assume that the interface requiring less “resources” is “preferable.” The design of an avatar interface requires the management and the development of humanoid animations and models as realistic as possible. If the animations are applied in real-time, the computational cost and the resources necessary to manage them may increase considerably. Moreover, since the field-of-view (FOV) of the AR mobile wearable devices is usually quite narrow (the one of the HoloLens glasses is around 35 °) and the size of the humanoid avatar is greater than the one of the abstract metaphors, users may face difficulties to visualize the real objects and the humanoid avatar at the same time. Watching only the arm and the pointing hand of the avatar may not be enough to detect the real objects. Furthermore, the relative huge dimensions of the virtual avatar may increase the occlusion problems related to the overlapping of the virtual objects on the real ones. Difficulties in perceiving the depth of the scene may decrease the overall quality of the avatar interface, also straining the users' sight. Taking into account the obtained results and the above considerations, it seems that the abstract metaphor-based interface should be preferred for managing remote maintenance operations.

Considering the results related to questions Q10-Q11-Q21-Q22 and from the analysis of the users' feedback, it has been possible to figure out that the audio channel has been fundamental in the interaction between the trainer and the trainee. This result seems to be confirmed from the analysis of the current state of art of the AR remote assistance systems. In fact, a remote assistance system is usually composed by both audio and video communication channels. Thus, it becomes important to understand which is the impact of the audio on the effectiveness of both interfaces and on the sense of presence. To achieve this goal, it has been decided to carry out some additional tests in no audio condition. Modality and results of the additional tests are presented in the following section.

### 5.1. Additional Tests

Some additional tests have been carried out to verify if the audio channel has lowered the differences between the two AR interfaces. The training procedure has been the same presented in section 4.1, but the feedback mechanism. In fact, users have no longer be supported by audio communications and thus a wizard feedback system has been employed. Users could inform an external collaborator if they had figured out the procedure and they could ask for repeating another time a specific animation.

Six new volunteers participated to this session test. They gave written informed consent in accordance with the Declaration of Helsinki and they have been divided in two groups (A and B). Although the reduced numbers of users do not allow obtaining statistically meaningful results, some observations can be deduced considering the M and SD values. The abstract AR interface has been considered more suitable than the avatar interface for the Efficiency and Learnability categories. Moreover, it has been deemed more gratifying than the avatar interface. Regarding the questions relative to the 3D assets, the abstract metaphors were found to be more useful to indicate the real objects and more effective to explain how to combine them. The possibility of visualizing the abstract metaphors and the real objects at the same time has allowed users to complete the task more efficiently. Concerning the questions relative to the “sense of presence,” results of Q5-Q16 and Q3-Q14 seem instead to suggest that in no audio condition the virtual avatar has been considered more suitable to express the presence of the trainer in the trainee environment.

Analyzing the results obtained from both tests, some final considerations are now presented. Since statistical results showing that one interface has performed better than the other have not be obtained, it is not possible to prove that the enhancement of the sense of presence, due to the virtual avatar, improves the performance of the users. Despite the fact that the virtual avatar seems to increase the sense of human-human collaboration in no audio condition, users have deemed more effective the abstract metaphors. Hence, it becomes necessary to investigate whether the sense of presence is unnecessary in industrial scenarios. Moreover, it becomes equally important to realize whether the avatar could be effective employed in tasks that require more complex physical gestures by the users, analyzing the interactions in audio and no audio conditions.

## 6. Conclusions and Future Works

In this paper, a comparison between two different AR interfaces has been proposed. The aim has been to investigate if a virtual human agent could improve the effectiveness of a training procedure and the sense of collaboration with a remote operator in an industrial context. The presented system is composed of a shared, mixed-reality environment which allows two users to interact using two distinct interfaces: an AR and an immersive VR interface. Specifically, a local operator, equipped with a wearable AR device, is able to receive support from a remote operator acting in an immersive virtual reality environment. Two distinct AR interfaces have been developed: in the first one, abstract metaphors have been used to explain the training procedure, whereas in the second one a virtual avatar has been presented to illustrate how to combine the required pieces. Since the obtained results are not statistically significant, it is not possible to determine if one interface is more efficient than the other. However, it is possible to infer some conclusions from this preliminary work: first of all, given the choice of the system to employ, it should be reasonable to develop the interface that requires less resources to be managed. Since abstract metaphors can be developed without having to take into account complex humanoid animations, that may be time and resources expensive, these might be preferred. Moreover, because of technological limitations, small virtual assets can be visualized more comfortably using wearable AR devices. Another relevant fact is that the audio communication channel plays a key role and it should be always integrated in such a system. Further tests should be carried out to statistically verify if audio instructions can completely replace any form of graphical hints. In no audio condition, the abstract interface has been considered by the testers more suitable for completing the maintenance task, making the sense of presence of the remote trainer unnecessary. Since these results have been obtained gathering only 6 questionnaires, additional tests are indeed necessary to evaluate the two AR interfaces. More complex scenarios will be also considered to verify whether more sophisticated animations can be more effective to express complex procedures. To improve the interaction of the trainer, it will be considered the adoption of external tracking devices that allow to represent faithful movements of the trainer. Human body tracking devices like the Vive^10^ or hand tracking systems such as the Leap Motion^11^ and the Manus^12^ gloves will be employed to enhance the VR interaction and therefore the AR interface. Results suggest that in industrial scenarios, the completion of the task is more important than the sense of collaboration. Further experiments will be conducted, involving more users in different tasks, in order to analyze the impact of significative physical displacements of the virtual agent and to verify if the sense of collaboration could have a greater impact on the effectiveness of the interface in more complex scenarios.

The addition of external tracking devices will be also taken into account to overcome the drawback of the pre-defined positions of the real pieces. The tracking procedure could be executed by the HoloLens itself or by an external vision system in order to avoid increasing the resources required by the wearable device. Moreover, the adoption of an external vision system will allow to continuously track the real objects independently from the point of view of the trainee, granting to correctly update the trainer environment and to automatically detect possible mistakes at the trainee side.

## Data Availability

All datasets generated for this study are included in the manuscript and/or the supplementary files.

## Author Contributions

FD and FM coordinated the designing of the system. Particular attention has been given in the synchronization of the AR and VR reference systems. The development of the multi-user system has been carried out by FD and DZ. An accurate analysis of the Unet API has been mandatory to understand how to efficiently align the different reference frames. FM and AS have carefully analyzed several possible training procedures that can be accomplished with an industrial manipulator. Finding a plausible industrial procedure has granted the possibility of testing the system in a realistic scenario. Test analysis has been carried out by AS and DZ. Several reliable considerations have been proposed that are indeed useful for the development of CMR systems.

### Conflict of Interest Statement

The authors declare that the research was conducted in the absence of any commercial or financial relationships that could be construed as a potential conflict of interest.

## Footnotes

^1^https://objecttheory.com/platform/

^2^https://www.microsoft.com/it-it/hololens

^3^https://assetstore.unity.com/packages/templates/systems/steamvr-plugin-32647

^4^https://docs.unity3d.com/Manual/UNet.html

^5^https://developer.vuforia.com/downloads/sdk

^6^https://github.com/grablab/openhand-hardware/tree/master/model%20t42

^7^https://www.eng.yale.edu/grablab/openhand/model%20t42/Fabrication%20-%20Model%20T42%201.0.pdf

^8^https://snapmaker.com/

^9^https://www.mixamo.com/#/

^10^https://www.vive.com/us/

^11^https://www.leapmotion.com/

^12^https://manus-vr.com/
