# Corrigendum: A Comparison Between Two Different Approaches for a Collaborative Mixed-Virtual Environment in Industrial Maintenance

**DOI:** 10.3389/frobt.2019.00051

**Published:** 2019-07-11

**Authors:** Francesco De Pace, Federico Manuri, Andrea Sanna, Davide Zappia

**Affiliations:** Dipartimento di Automatica e Informatica, Politecnico di Torino, Turin, Italy

**Keywords:** augmented reality, virtual reality, mixed-reality, shared-reality, collaborative environment, interfaces, industry 4.0

In the original article, there was a mistake in Figure 3, Figure 7, and Figure 8 as published. Due to non-disclosure agreement complications, three figures of the paper should be replaced. The paper's authors apologize for these errors and they ensure that the main subject of the figures has not changed, only a few adjustments have been made to the background and very few details have been cut off from the images. These adjustments do not compromise the reading of the article in any way. The three figures are the following (notice that the same name conventions has been used). The original article has been updated.

**Figure 3:**
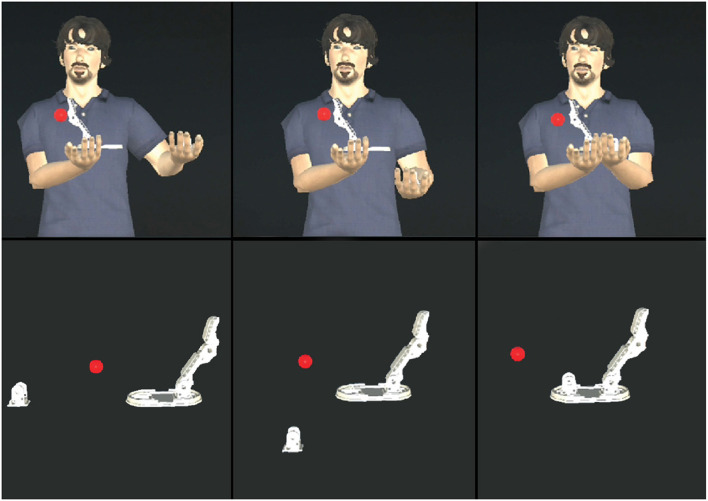
First row: from left to right, one of the assembly animations played by the virtual avatar. Second row: the same animation played without the virtual avatar in the abstract AR interface.

**Figure 7:**
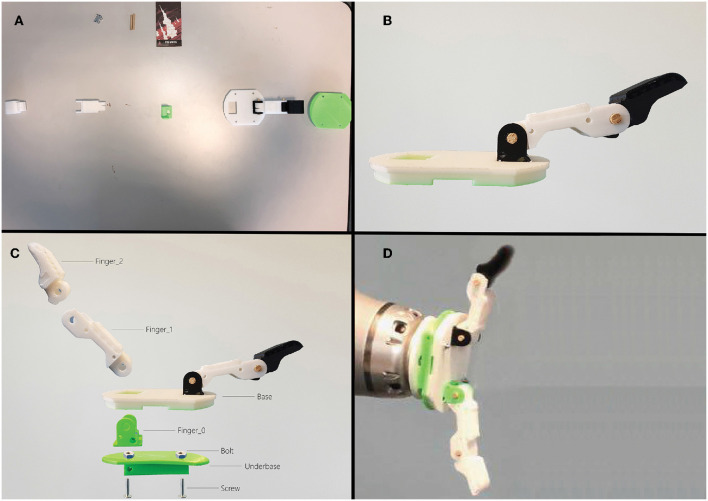
Top-left image **(A)**: the initial configuration. Top-right image **(B)**: the finger already assembled. Bottom-left image **(C)**: the renamed hand pieces. Bottom-right image **(D)**: the complete hand placed at the end-effector position.

**Figure 8:**
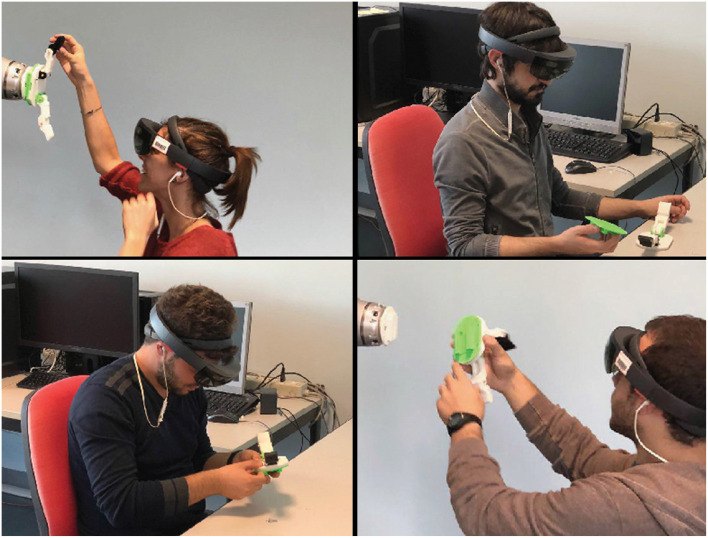
Users evaluating the multi-users mixed reality environment.

